# Lack of *Bcl10* mutations in testicular germ cell tumours and derived cell lines

**DOI:** 10.1038/sj.bjc.6690563

**Published:** 1999-07

**Authors:** E M van Schothorst, S Mohkamsing, R J H L M van Gurp, J W Oosterhuis, P T van der Saag, L H J Looijenga

**Affiliations:** Laboratory for Experimental Patho-Oncology, Pathology/Daniel den Hoed Cancer Center, Josephine Nefkens Institute, University Hospital Rotterdam, PO Box 1738, 3000 DR Rotterdam, The Netherlands; Netherlands Institute of Developmental Biology, Hubrecht Laboratory, Uppsalalaan 8, 3584 VT Utrecht, The Netherlands

**Keywords:** testicular cancer, *Bcl10*, mutation-analysis

## Abstract

Aberrations within *Bcl10*, a gene involved in execution of apoptosis, has most recently been found in a variety of cancers, including cell lines of testicular germ cell tumours of adolescents and adults (TGCTs). To study this in more detail, we screened exons 2 and 3 of this gene for mutations in a larger series of cell lines as well as primary TGCTs by single-strand conformation polymorphism and endonuclease restriction analysis. Because no aberrations were detected, we conclude that inactivation of *Bcl10* by mutation is at least far less important in the development of TGCTs than proposed. © 1999 Cancer Research Campaign


*British Journal of Cancer* (1999) **80**(10), 1571–1574
© 1999 Cancer Research Campaign
Article no. bjoc.1999.0563

### Lack of *Bcl10* mutations in testicular germ cell tumours and derived cell lines

**EM van Schothorst[1,2], S Mohkamsing[1], RJHLM van Gurp[1], JW Oosterhuis[1], PT van der Saag[2] and LHJ Looijenga[1]**

[1]Laboratory for Experimental Patho-Oncology, Pathology/Daniel den Hoed Cancer Center, Josephine Nefkens Institute, University Hospital Rotterdam, PO Box 1738, 3000 DR Rotterdam, The Netherlands; [2]Netherlands Institute of Developmental Biology, Hubrecht Laboratory, Uppsalalaan 8, 3584 VT Utrecht, The Netherlands

**Summary** Aberrations within *Bcl10*, a gene involved in execution of apoptosis, has most recently been found in a variety of cancers, including cell lines of testicular germ cell tumours of adolescents and adults (TGCTs). To study this in more detail, we screened exons 2 and 3 of this gene for mutations in a larger series of cell lines as well as primary TGCTs by single-strand conformation polymorphism and endonuclease restriction analysis. Because no aberrations were detected, we conclude that inactivation of *Bcl10* by mutation is at least far less important in the development of TGCTs than proposed.

**Keywords:** testicular cancer; *Bcl10*; mutation-analysis

One possible event in the development of cancer is a disturbed induction of apoptosis, i.e. programmed cell death. This will lead to an enlarged population of target cells for the subsequent events, finally leading to the fully malignant phenotype. The classical example of this mechanism, eventually resulting in follicular B-cell non-Hodgkin's lymphomas, is the outcome of a translocation of chromosome 18 band q21 and chromosome 14 band q32. This translocation places *Bcl2* under control of the *IgH* promoter, leading to overexpression, and subsequent inhibition of apoptosis (Tsujimoto et al, 1984). More recently, an alternative and intriguing mechanism has been reported in B-cell lymphomas of mucosa-associated lymphoid tissue (MALT lymphomas). *Bcl10*, a gene mapped to chromosome 1 band P22, encodes a protein with caspase activity, which is involved in the execution of the apoptotic machinery. Translocation of this gene to chromosome 14 band q32, as has been found in MALT lymphomas, results in a disrupted caspase activity, thereby preventing apoptosis (Willis et al, 1999). Interestingly, aberrations within this gene, like point-mutations and small deletions, were also found in a variety of cancers, including colon cancers, mesotheliomas and testicular germ cell tumours of adolescents and adults (TGCTs). In total, 87 cell lines were screened for aberrations, 81 on genomic DNA level, and six using cDNA clones. The high incidence of aberrations in *Bcl10* found, for example, in three out of three TGCT-derived cell lines, suggests that inactivation of this gene is a general and crucial mechanism in the pathogenesis of different types of malignancies.

TGCTs are the most common malignancy in young Caucasian males aged between 15 and 45 years (Swerdlow, 1998), and the incidence is still increasing. It has generally been accepted that these cancers originate from an early germ cell, of which the malignant counterpart is referred to as carcinoma in situ (Skakkebæk, 1972; Jørgensen et al, 1997). Histologically and clinically, TGCTs are divided into non-seminomatous-TGCTs (NS-TGCTs) and seminomas (Ulbright, 1993). While seminomas mimic carcinoma in situ, the NS-TGCTs reflect embryonal development to a certain extent. They can be composed of embryonal carcinoma, being the undifferentiated cells, or teratoma, mimicking somatic differentiation. In addition, the extra-embryonal elements yolk sac tumour and choriocarcinoma can be found. Total DNA content analysis showed that all TGCTs, including carcinoma in situ, are aneuploid (Oosterhuis et al, 1989; De Graaff et al, 1992). Karyotyping, allelic imbalances analysis and, more recently, comparative genomic hybridization revealed a relatively consistent pattern of gains and losses (Mathew et al, 1994; Van Echten-Arends et al, 1995; Sandberg et al, 1996; Looijenga and Oosterhuis, 1999, and references cited therein). For example, loss of (part of) the short arm of chromosome 1 has been found both in seminomas and NS-TGCTs, suggesting the presence of a tumour suppressor gene.

To investigate the suggested role of *Bcl10* in the development of TGCTs more extensively, we studied a series of derived and related cell lines, as well as primary tumours for genomic aberrations within exon 2 and 3 of this gene. Therefore we applied the technique of single-strand conformation polymorphism (SSCP) analysis, as well as a more straightforward analysis for a number of reported aberrations by restriction endonuclease digestion analysis.

### MATERIALS AND METHODS

The NS-TGCT-derived cell lines Tera-1, Tera-2, GCT44, 2102 EP, NTera-2, GCT27 and NCCIT, were included. In addition, the cell lines Haz and Bos, derived from two extragonadal germ cell tumours, were studied (Sinke et al, 1994; Van Echten-Arends et al, 1995), as well as the cell line S2, of which the origin is still a matter of debate (Von Keitz et al, 1994; Andrews et al, 1996). The mesothelioma-derived cell line M25 was also investigated (Versnel et al, 1989). The cell lines were cultured as described

*Received 22 April 1999*
*Accepted 26 April 1999*

*Correspondence to:* LHJ Looijenga

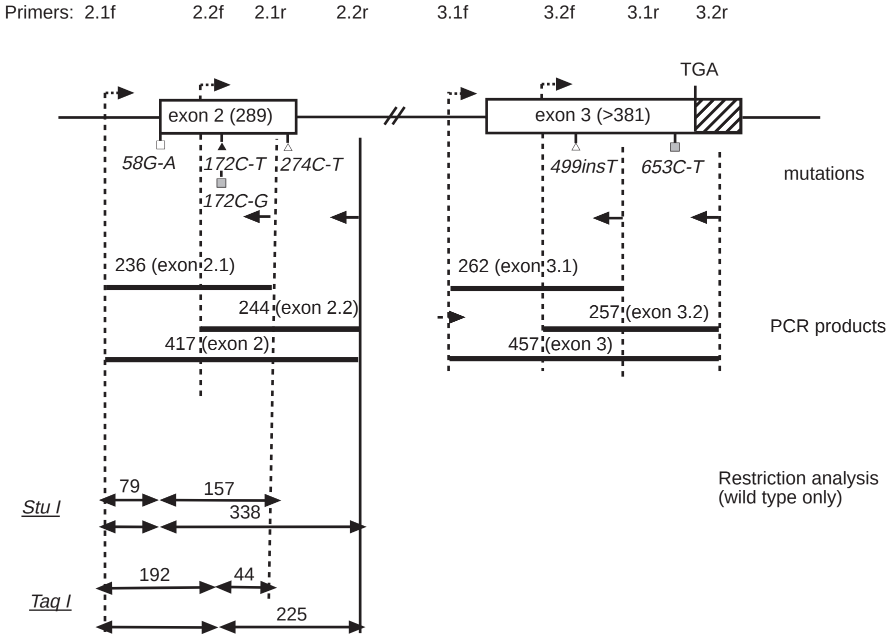

**Figure 1** Screening strategy for aberrations within exons 2 and 3 of the *Bcl10* gene. Exons are denoted at the top by rectangles (not on scale; size in bp is given between brackets); the primers are represented by arrows and reported mutations either by triangle (truncation) or square (mis-sense). The code used for DNA origin is as follows: white = Tera-2 (cDNA mutations, one within the *Stu*I recognition site), grey = Tera-1 (within the *Taq*I recognition site), and black = GCT44 (within *Taq*I recognition site). The 3′ untranslated region is given by a hatched box. Horizontal bold bars represent the amplified fragments, as well as fragments obtained after restriction endonuclease digestion (either *Stu*I or *Taq*I) of the wild-type sequence (sizes in bp). The amplified exon 2.1 fragment is 236 bp in length instead of the 200 bp as indicated before (Willis et al, 1999). This is confirmed by analysis of the length of the fragment obtained by primers 2.1f–2.2r, giving rise to a 417 bp fragment and not a 381 bp as deduced from the data by Willis et al (1999)

before (Andrews et al, 1996). Four primary testicular seminomas and four NS-TGCTs, with the histology of pure embryonal carcinoma, pure teratoma, pure yolk sac tumour and mixed containing embryonal carcinoma, teratoma and yolk sac tumour, were investigated. In addition, one gonadoblastoma (reviewed in Hussong et al, 1997), one Leydig cell tumour and one spermatocytic seminoma (reviewed in Rosenberg et al, 1998) were studied. The tumours were snap-frozen upon orchidectomy. Peripheral blood DNA of three unrelated individuals were included in every experiment as control, and all experiments were done at least twice. DNA was isolated from the in vitro cultured cell lines, peripheral blood and tumours using standard procedures as described before (Sambrook et al, 1989).

Exons 2 and 3 were screened for the presence of aberrations on genomic DNA level by SSCP and restriction endonuclease digestion analysis on polymerase chain reaction (PCR)-amplified fragments. Positions within the genomic sequence of the primers used for amplification are schematically represented in Figure 1 and are identical to those described (Willis et al, 1999). Amplification was done in a total volume of 25 µl of buffer (10 mM Tris–HCl pH 8.8, 50 mM potassium chloride, 0.1% Triton X-100, 2 mM magnesium chloride; 5 pmol of each primer; 0.2 mM dNTP and 1 U *Taq* (Promega, Madison, WI, USA)), containing 25 ng high molecular weight DNA. The PCR programme consisted of an initial denaturation of 4 min at 94°C, 35 cycles of 1 min at 94°C denaturation, 1 min at 60°C annealing and 1 min at 72°C elongation, respectively, followed by 6 min at 72°C.

SSCP was carried out at 4°C on a 6% non-denaturing polyacrylamide gel using radioactive labelled PCR fragments. Briefly, 1 µl of α-$^{32}$P-dATP (ICN, Zoetermeer, The Netherlands) was added to the PCR-mix and, after amplification, 25 µl sequencing loading buffer was added. After denaturation, 4 µl was loaded on the gel and run for 5 h at 60 Watt, whereafter the gel was dried. BioMax film (Kodak, Rochester, NY, USA) was exposed overnight at –80°C. To exclude misinterpretation of the SSCP patterns due to the presence of double-stranded DNA fragments, non-denatured PCR fragments were included.

To confirm the absence of specific aberrations, endonuclease restriction digestion analysis was done on amplification products of (part of) exon 2 by the primer sets as given in Figure 1 (either exon 2 or exon 2.1, which is the same primer set as used for SSCP analysis; see above). Therefore 5 µl PCR product was digested with 1 unit of *Stu*I or *Taq*I according to the manufacturer's instructions (Pharmacia Biotech, Uppsala, Sweden) in a total volume of 10 µl. After digestion, the samples were run on a 2% agarose gel stained with ethidium bromide and analysed using the IS-500 digital imaging system and AlphaImager 2000 (Alpha Innotech, San Leandro, CA, USA). Aberrations within the recognition sites of these specific restriction endonucleases will result in the presence of undigested fragments.

Direct sequencing of amplified fragments of exon 2 and 2.1 using end-labelled primers 2.1r and 2.2r (Figure 1) was performed using the cycle-sequencing kit (Perkin-Elmer Cetus, Norwalk, CT, USA).

### RESULTS

To screen DNA isolated from NS-TGCT-derived and related cell lines, as well as primary testicular tumours, including both seminomas and NS-TGCTs, for aberrations within exon 2 and 3 of the *Bcl10* gene, initially SSCP was performed. For both exons, two

 © 1999 Cancer Research Campaign

2.1f

. . aagact gccaact aat agt cacgt at gt gat t t t ct t agat gt t aaaaat exon 2

aacat t gaaccat at t t t at gct t t t agGCCTTAGAAAATTTACGTGTA. .

58

**Figure 2** Sequence analysis of the 3-prime region of intron 1 of *Bcl10*. Direct sequencing of amplified products 2 and 2.1 revealed the following intronic sequence (lower case); the forward primer 2.1f is denoted with an arrow (above), and the exon 2 sequence (GenBank AJ006288) in uppercase letters with the nucleotide numbering underneath for orientation purposes. DNA samples analysed, i.e. Tera-1 and CGT44, only showed the wild-type sequence

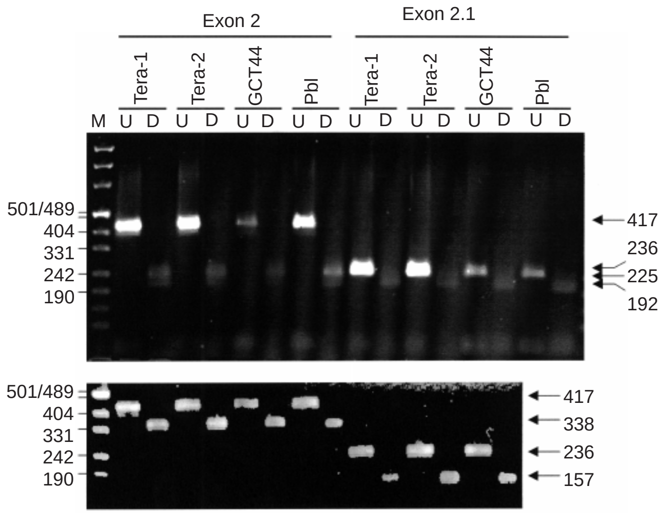

**Figure 3** Representative results of the restriction enzyme digestion analysis of the amplification products of exon 2 of the *Bcl10* gene with *Taq*I and *Stu*I. Using exon 2 amplified fragments (2 and 2.1) and subsequently restriction analysis with the enzymes *Taq*I (top) and *Stu*I (bottom), all samples showed an identical pattern (sizes are given on the right side with arrows) as found in the control samples denoted as Pbl (= DNA isolated from peripheral blood of healthy individuals). Abbreviations used: U = undigested PCR products; D = digested PCR products. M: DNA size marker pUC Mix (2 µl (100 ng); Fermentas, Vilnius, Lithuania); sizes are given on the left side sets of primers were used, resulting in two overlapping fragments, designated 2.1, 2.2, 3.1 and 3.2 respectively (see Figure 1). The expected sizes are indicated in the Figure. In contrast to the reported length of 200 bp (Willis et al, 1999), the 2.1 fragment is found to be 236 bp, both in controls, cell lines and tumours. Direct sequencing of the amplification products demonstrated that a 54 bp (and not 18 bp) intronic sequence separates the 2.1 forward primer and exon 2 (see Figure 2 for the additional sequence). All cell lines and tumours showed a similar SSCP pattern, identical to the pattern detected in the control samples (not shown). The presence of double-stranded fragments, possibly leading to misinterpretation, was excluded within the protocol. Within the series of NS-TGCT-derived cell lines screened in this study, Tera-1 and GCT44 were also investigated (see Materials and Methods), and were not found to contain aberrations on the genomic DNA level, although these cell lines were shown to contain mutations in the original study by Willis et al (1999; see Fig. 1).

To exclude that the presence of specific aberrations within exon 2 remained undetected by SSCP, as can be the case (Landegren et al, 1998), we additionally performed a more direct analysis. Therefore restriction endonuclease digestions were performed on the amplification products of exon 2 (both 2 and 2.1) using *Stu*I and *Taq*I (see Figure 1). These restriction endonucleases digest the wild-type sequence, resulting in fragments of respectively 79 + 157 and 79 + 338 bp for *Stu*I, depending on the primer sets applied, and 192 + 44 and 192 + 225 bp for *Taq*I respectively. In case of a mutation within either the *Stu*I or *Taq*I recognition site, an undigested fragment of 236 or 417 bp will be found. A disrupted *Stu*I recognition site can be expected for Tera-2, although this mutation was only reported in a cDNA clone. None of the cell lines and tumours tested in this study showed the presence of undigested fragments after *Stu*I and *Taq*I digestion, indicating the absence of mutations within these sequences, of which representative examples are illustrated in Figure 3. These results were confirmed by direct sequencing of the amplification products (data not shown). Moreover, we also studied the mesothelioma cell line M25, found to contain a deletion of 16 bp within exon 3. In our hands, PCR showed the wild-type length of this fragment in this particular cell line (not shown).

### DISCUSSION

Apoptosis is found to be crucial for normal development and maintenance of an organism. In this process, caspase (‘cysteinyl aspartate-specific proteinases’) activity is important for proper execution. Several caspases are identified so far (Salvesen and Dixit, 1997). In addition to its importance during normal development and maintenance, a disturbed apoptosis has also been identified as a possible event in the development of cancer. This can be the result of activation of apoptosis-inhibiting genes, like *Bcl2*, or, more recently, also due to inactivation of apoptosis-inducing genes. Disruption of the caspase activity of *Bcl10* has most

© 1999 Cancer Research Campaign 

recently been found in MALT lymphomas (Willis et al, 1999). In addition, this study also identified mutations within exons 2 and 3 of this gene in a variety of cancers of different origin, including three out of three NS-TGCT-derived cell lines. It is of note, that we suggested inhibition of apoptosis being one of the mechanisms involved in the pathogenesis of TGCTs, i.e. lack of induction of apoptosis of the precursor cell of this cancer (Looijenga and Oosterhuis, 1999). Moreover, we showed that *RAS* mutation or amplification leads to an enhanced in vitro survival of seminoma cells, related to a reduced sensitivity for apoptosis upon disruption of the micro-environment (Oosterhuis et al, 1998; Looijenga and Oosterhuis, 1999).

To study the role of *Bcl10* in the pathogenesis of TGCTs in more detail, we performed in principle a similar approach as Willis et al (1999). DNA of a series of cell lines were investigated by SSCP using the same primer sets as reported before. However, no indications for the presence of aberrations within exons 2 and 3 were obtained in our hands. The presence of independent and multiple controls without denaturation before SSCP prevented misinterpretation of the data due to double-stranded fragments in the samples under investigation. Besides the cell lines, a series of primary TGCTs also showed no aberrant bands by SSCP. In fact, even no aberrant migration patterns of the fragments derived from the cell lines Tera-1 and GCT44 were observed, in spite of the results reported before (Willis et al, 1999). In addition, the restriction endonuclease digestion analysis for specific sequence abnormalities, also showed no undigested fragment, i.e. only wild-type alleles were amplified, which was confirmed by direct sequencing. There is no reason to assume the inability of amplification of mutant alleles, because of the use of flanking sets of primers (see Figure 1). Because in the initial study the majority of aberrations were detected on the genomic DNA level, we limited our survey to genomic DNA analysis. The formerly reported mutations in the cDNA clones of the Tera-2 cell line could not be confirmed by us on genomic DNA.

Theoretically, the discrepancies between the study of Willis et al (1999) and our results (this study) could be due to the extended in vitro culturing of the different cell lines, both from NS-TGCT- and mesothelioma-origin. Indeed, we have found other differences between clones derived from the same parental cell line, which were cultured in different laboratories for extended periods of time (Looijenga et al, 1997). However, assuming that inactivation by aberrations at the genomic DNA level of *Bcl10* is important in the development of TGCTs, which could be lost upon in vitro growth, these aberrations should have been present in primary TGCTs. Although we only screened a limited number of seminomas and NS-TGCTs, no indications were obtained for the presence of mutations within this gene, not with SSCP nor by restriction endonuclease digestion analysis.

Although still of putative interest in general, our results specifically question the role of inactivation of *Bcl10* in the development of TGCTs. We conclude that lack of induction of apoptosis due to loss of caspase activity by genomic events in TGCTs is not involved in the majority of cases, if at all.

### ACKNOWLEDGEMENTS

This project is financially supported by the Dutch Cancer Society (JWO and LL, and grant 97 1516 for EMvS and SM). We kindly thank Dr MA Versnel, Department of Immunology, Erasmus University Rotterdam, The Netherlands for providing the mesothelioma cell line.

### REFERENCES

Andrews PW, Casper J, Damjanov I, Duggan-Keen M, Giwercman A, Hata J-i, Von Keitz A, Looijenga LHJ, Oosterhuis JW, Pera M, Sawada M, Schmoll H-J, Skakkebæk NE, Van Putten W and Stern P (1996) A comparative analysis of cell surface antigens expressed by cell lines derived from human germ cell tumors. *Int J Cancer* **66**: 806–816

De Graaff WE, Oosterhuis JW, De Jong B, Dam A, Van Putten WLJ, Castedo SMMJ, Sleijfer DT and Schraffordt Koops H (1992) Ploidy of testicular carcinoma in situ. *Lab Invest* **66**: 166–168

Hussong J, Crussi FG and Chou PM (1997) Gonadoblastoma: immunohistochemical localization of Mullerian-inhibiting substance, inhibin, WT-1, and p53. *Mod Pathol* **10**: 1101–1105

Jørgensen N, Müller J, Jaubert F, Clausen OP and Skakkebæk NE (1997) Heterogeneity of gonadoblastoma germ cells: similarities with immature germ cells, spermatogonia and testicular carcinoma in situ cells. *Histopathology* **30**: 177–186

Landegren U, Nilsson M and Kwok PY (1998) Reading bits of genetic information: methods for single-nucleotide polymorphism analysis. *Genome Res* **8**: 769–776

Looijenga LHJ and Oosterhuis JW (1999) Pathogenesis of testicular germ cell tumors. *Rev of Reprod* **4**: 90–100

Looijenga LHJ, Gillis AJM, Van Gurp RJHLM, Verkerk AJMH and Oosterhuis JW (1997) X inactivation in human testicular tumors. XIST expression and androgen receptor methylation status. *Am J Pathol* **151**: 581–590

Mathew S, Murty VVVS, Bosl GJ and Chaganti RSK (1994) Loss of heterozygosity identifies multiple sites of allelic deletions on chromosome 1 in human male germ cell tumors. *Cancer Res* **54**: 6265–6269

Oosterhuis JW, Castedo SMMJ, De Jong B, Cornelisse CJ, Dam A, Sleijfer DT and Schraffordt Koops H (1989) Ploidy of primary germ cell tumors of the testis. Pathogenetic and clinical relevance. *Lab Invest* **60**: 14–20

Oosterhuis JW, Gillis AJM and Looijenga LHJ (1998) In vitro survival, RAS mutations, apoptosis and activation of the SAPK-pathway in human seminoma cells. In: *Germ Cell Tumours IV*, Jones WG, Appleyard I, Harnden P and Joffe JK (eds), pp. 51–58. John Libbey: London

Rosenberg C, Mostert MC, Bakker Schut T, Van de Pol M, Van Echten-Arends J, De Jong B, Raap T, Tanke H, Oosterhuis JW and Looijenga LHJ (1998) Chromosomal constitution of human spermatocytic seminomas: comparative genomic hybridization supported by conventional and interphase cytogenetics. *Genes Chromosomes Cancer* **23**: 286–291

Salvesen GS and Dixit VM (1997) Caspases: intracellular signalling by proteolysis. *Cell* **91**: 443–446

Sambrook J, Frisch EF and Maniatis T (1989) *Molecular Cloning: a Laboratory Manual*. Cold Spring Harbor Laboratory Press: New York

Sandberg AA, Meloni AM and Suijkerbuijk RF (1996) Reviews of chromosome studies in urological tumors. 3. Cytogenetics and genes in testicular tumors. *J Urol* **155**: 1531–1556

Sinke RJ, Olde Weghuis D, Suijkerbuijk RF, Tanigami A, Nakamura Y, Larsson C, Weber G, De Jong B, Oosterhuis JW, Molenaar WM and Geurts van Kessel A (1994) Molecular characterization of a recurring complex chromosomal translocation in two human extragonadal germ cell tumors. *Cancer Genet Cytogenet* **73**: 11–16

Skakkebæk NE (1972) Possible carcinoma-in-situ of the testis. *Lancet* 516–517

Swerdlow AJ (1998) New research in testicular cancer epidemiology. In: *Germ Cell Tumours IV*, Jones WG, Appleyard I, Harnden P and Joffe JK (eds), pp. 3–8. John Libbey: London

Tsujimoto Y, Yunis J, Onorato-Showe L, Erikson J, Nowell PC and Croce CM (1984) Molecular cloning of the chromosomal breakpoint of B-cell lymphomas and leukemias with the t(11;14) chromosome translocation. *Science* **224**: 1403–1406

Ulbright TM (1993) Germ cell neoplasms of the testis. *Am J Surg Pathol* **17**: 1075–1091

Van Echten-Arends J, Oosterhuis JW, Looijenga LHJ, Wiersma J, te Meerman G, Schraffordt Koops H, Sleijfer DT and De Jong B (1995) No recurrent structural abnormalities in germ cell tumors of the adult testis apart from i(12p). *Genes Chromosomes Cancer* **14**: 133–144

Versnel MA, Hoogsteden HC, Hagemeijer A, Bouts MJ, van der Kwast TH, Delahaye M, Schaart G and Ramaekers FC (1989) Characterization of three human malignant mesothelioma cell lines. *Cancer Genet Cytogenet* **42**: 115–128

Von Keitz AT, Riedmiller H, Neumann K, Gutschank W and Fonatsch C (1994) Establishment and characterization of a seminoma cell-line (S2). *Invest Urol* **5**: 28–31

Willis TG, Jadayel DM, Du MQ, Peng H, Perry AR, Abdul-Rauf M, Price H, Karran L, Majekodunmi O, Wlodarska I, Pan L, Crook T, Hamoudi R, Isaacson PG and Dyer MJ (1999) Bcl10 is involved in t(1;14)(p22;q32) of MALT B cell lymphoma and mutated in multiple tumor types. *Cell* **96**: 35–45

*© 1999 Cancer Research Campaign*
